# A systematic review of the role of human papilloma virus (HPV) testing within a cervical screening programme: summary and conclusions

**DOI:** 10.1054/bjoc.2000.1375

**Published:** 2000-08-16

**Authors:** J Cuzick, P Sasieni, P Davies, J Adams, C Normand, A Frater, M van Ballegooijen, E van den Akker-van Marle

**Affiliations:** Department of Mathematics, Statistics and Epidemiology, Imperial Cancer Research Fund, London, UK; BMI Health Services, London, UK; Department of Public Health and Policy, Health Services Research Unit, London School of Hygiene and Tropical Medicine, UK; Faculty of Medicine and Health Sciences, Erasmus Universiteit, Rotterdam, The Netherlands

**Keywords:** HPV testing, cervical screening, systematic review

## Abstract

A systematic review of the available evidence on the role of HPV testing in cervical screening has been published by the Health Technology Assessment Committee of the UK Department of Health. The review summarized relevant data on testing methods, natural history, and prevalence of the virus in different disease groups. Cost-effectiveness modelling was undertaken. Ten major conclusions were reached and are reported here. The key conclusions were that HPV testing was more sensitive than cytology, but that there were concerns about specificity, especially in young women. The increased sensitivity led to a recommendation that HPV testing be introduced on a pilot basis for women with borderline and mild smears. HPV testing has great potential as a primary screening test, but large trials are needed to properly evaluate this application and to determine if its introduction can reduce invasive cancer rates. There is an urgent need to undertake a large trial of HPV testing in conjunction with other new technologies (liquid-based cytology and computer-assisted cytology reading) to determine the best way to integrate them into ongoing screening programmes. A range of issues including the age to start and stop screening, the appropriate screening interval, the role of self-sampling for HPV testing and the choice of primary test (HPV and/or cytology) require further evaluation. © 2000 Cancer Research Campaign


*British Journal of Cancer* (2000) **83**(5), 561–565
© 2000 Cancer Research Campaign
doi: 10.1054/ bjoc.2000.1375, available online at http://www.idealibrary.com on IDEAL®

### A systematic review of the role of human papilloma virus (HPV) testing within a cervical screening programme: summary and conclusions

**J Cuzick[1], P Sasieni[1], P Davies[2], J Adams[1], C Normand[3], A Frater[3], M van Ballegooijen[4] and E van den Akker-van Marle[14]**

[1]Department of Mathematics, Statistics and Epidemiology, Imperial Cancer Research Fund, London, UK; [2]BMI Health Services, London, UK; [3]Department of Public Health and Policy, Health Services Research Unit, London School of Hygiene and Tropical Medicine, UK; [4]Faculty of Medicine and Health Sciences, Erasmus Universiteit, Rotterdam, The Netherlands

**Summary** A systematic review of the available evidence on the role of HPV testing in cervical screening has been published by the Health Technology Assessment Committee of the UK Department of Health. The review summarized relevant data on testing methods, natural history, and prevalence of the virus in different disease groups. Cost-effectiveness modelling was undertaken. Ten major conclusions were reached and are reported here. The key conclusions were that HPV testing was more sensitive than cytology, but that there were concerns about specificity, especially in young women. The increased sensitivity led to a recommendation that HPV testing be introduced on a pilot basis for women with borderline and mild smears. HPV testing has great potential as a primary screening test, but large trials are needed to properly evaluate this application and to determine if its introduction can reduce invasive cancer rates. There is an urgent need to undertake a large trial of HPV testing in conjunction with other new technologies (liquid-based cytology and computer-assisted cytology reading) to determine the best way to integrate them into ongoing screening programmes. A range of issues including the age to start and stop screening, the appropriate screening interval, the role of self-sampling for HPV testing and the choice of primary test (HPV and/or cytology) require further evaluation. © 2000 Cancer Research Campaign

**Keywords:** HPV testing; cervical screening; systematic review

We have recently completed a 200-page report entitled ‘A Systematic Review of the Role of Human Papilloma Virus (HPV) Testing within a Cervical Screening Programme’ (Cuzick et al, 1999b) for the Health Technology Assessment Committee of the British Department of Health. As this document is not easily available, here we report the main results of that review including the conclusions and recommendations.

There is little doubt that well-organized cytology-based screening programmes for cervical cancer have been effective in reducing cancer incidence and preventing premature deaths. This is especially true of those that have good quality assurance. Potential reductions in disease of 60–90% are possible in the three years after screening (IARC, 1986; Sasieni et al, 1996). The importance of good coverage and quality control is demonstrated by the accelerated decline in mortality in England and Wales following the changes implemented in 1988 (Sasieni et al, 1995), addressing problems identified in the early 1980s (ICRF, 1984, 1986). However, a recent audit (Sasieni et al, 1996) found that 47% of fully invasive cancers in women under the age of 70 occurred despite an apparently adequate screening history, suggesting problems with the sensitivity of the test. Further progress in reducing the disease burden is therefore likely to come from a combination of measures to extend the coverage of screening, and more accurately identify women with precursor lesions.

*Received 19 January 2000*
*Revised 1 May 2000*
*Accepted 15 June 2000*

*Correspondence to:* J Cuzick

The sensitivity of cytology is limited by sampling error, in which the abnormal cells do not get placed on the smear, and reading error, where a few abnormal cells are not identified among the multitude of normal cells that are also present in a well-taken cervical smear. Sensitivities for cytology of only 40–80% for high-grade cervical intraepithelial neoplasia (CIN II/III) have been reported (Reid et al, 1991; Cox et al, 1995; Cuzick et al, 1995). Furthermore, cytologic screening is poor at detecting glandular lesions or adenocarcinoma, which account for a growing number of cervical cancers (Kjaer and Brinton, 1993; Vizcaino, et al, 1998). Cytology also has problems with specificity, and the screening programme is overburdened by borderline and mildly dyskaryotic smears, which are costly to follow-up, cause anxiety to the women concerned, but have low predictive value for high-grade pathology (Shafi, 1994; Raffle et al, 1995).

The causal association between infection with certain HPV types and the development of cervical cancer is now beyond reasonable dispute. The epidemiological data supporting this assertion include reports that HPV DNA can be recovered from over 95% of all cervical tumours (Bosch et al, 1995; Walboomers et al, 1999; Cuzick et al, 2000), and that women infected with oncogenic HPV types have relative risks of 40–180 for the development of high-grade cervical disease (IARC, 1995; Olsen et al, 1995). Additionally, molecular studies have identified mechanisms by which high-risk HPV types contribute to carcinogenesis. The World Health Organization and the International Agency for Research on Cancer have officially designated HPVs 16 and 18 as carcinogenic agents. Even higher relative risks (100–500) are reported for persistent HPV infection, which appears to be the key step in cervical carcinogenesis (Nobbenhuis et al, 1999).

A plateau of what can be achieved by conventional cytology is now being reached, and the fundamental importance of HPV in the aetiology of cervical cancer has been clearly demonstrated. There is therefore much interest in the use of HPV testing to improve both the effectiveness and cost-effectiveness of cervical screening. Thus, it is timely to consider the role of human papilloma virus (HPV) testing within the cervical screening programme and to review research into its potential implementation.

### METHODS

Eight databases were searched, producing a total of about 2100 papers. Additional references were sought by scanning the citations of review articles and books devoted to HPV. Ongoing and unpublished studies were identified, but their results are not included.

The main objectives of the review were as follows.

1. To evaluate the available data concerning the role of HPV testing:
   a. in primary screening, either alone or as an adjunct to cytology;
   b. to improve the management of women with low-grade cytologic abnormalities;
   c. to improve the accuracy of follow-up after treatment of preinvasive or early invasive lesions.
2. To review the methods available for HPV testing and determine their appropriateness for widespread implementation.
3. To determine what future research is required to obtain more reliable answers about its use in screening.

An attempt was made to answer a series of questions formulated at the outset.

Papers were divided into broad categories and initially screened by title and abstract using predefined criteria. Complete copies of papers not rejected were obtained, and data were abstracted. Abstractions were done by one author and checked by another. Tabular, graphical and textual methods were used to synthesize the data.

In addition, results from important subsequent publications have also been noted.

### RESULTS

### Testing methodology

A range of approaches have been used to detect HPV in smear material with widely differing results. The most thoroughly studied methods are now being superseded by newer methods that offer better sensitivity, specificity and reproducibility and are easier to perform. However, many of the most relevant studies are just beginning to reach the literature, and most of the large studies related to screening are still ongoing with at most only preliminary reports available. Currently, two consensus primer PCR systems – the MY09/11 and the GP5+/6+ pairs – and the second-generation Hybrid Capture system (HC-II) with high risk probes would seem to be the methods of choice (Manos et al, 1989; de Roda Husman et al, 1995; Peyton et al, 1998). These three methods all have high absolute sensitivity for detecting oncogenic viruses and have the potential for automation. Developments in the form of second-stage assays may help improve specificity without substantially reducing sensitivity.

### Natural history

HPV is a sexually transmitted disease with peak prevalence in the age group 20–24 years which gradually declines up to about age 40–45 years, but then may begin to increase slowly again (Herrero et al, 2000). Most infections are transient, with a median duration of at most 12 months, and pose no risk of cervical neoplasia: only the 10–20% that remain persistent are of concern (Ho et al, 1995; Remmink et al, 1995; Nobbenhuis et al, 1999). Evidence of infection, either by serology in stored blood samples or by viral DNA in fixed archival specimens, is found many years before serious disease is present, and indicates that infection precedes disease (Walboomers et al, 1995; Lehtinen et al, 1996; Dillner et al, 1997). Detection of HPV DNA in the absence of cytological abnormalities can also indicate presence of high-grade cervical intraepithelial neoplasia (CIN) which was missed by cytology (Cuzick et al, 1995). Women with minor cytological abnormalities who test negative for oncogenic HPV have a low risk of developing high-grade CIN within 3 years. Little is known about the progressive potential of CIN detected by HPV testing, but missed by cytology or vice versa.

### Prevalence

The review was limited to studies using consensus PCR or Hybrid Capture, as other tests are much less reliable and give results that are not comparable. With modern tests, over 95% of all cancers are HPV positive (Bosch et al, 1995; Walboomers et al, 1999; Cuzick et al, 2000) and 75–95% of high-grade CIN lesions are associated with a positive HPV test on exfoliated cells (de Roda Husman et al, 1995; Herrington et al, 1995; Burger et al, 1996; Clavel et al, 1999; Cuzick et al, 1999). These and other comparative studies have shown that HPV testing has a greater sensitivity for CIN II/III than cytology. However, greater variability in the HPV positivity rate in 'normal' populations is seen, leading to concern about specificity especially in younger women. This variability reflects a number of factors, including age, extent of sexual exposure, previous disease, and type of assay used. Overall the positivity rate of tests for high-risk HPV types in women not known to have CIN was 13% ranging from 10% for PCR using GP5/6 primers (de Roda Husman et al, 1995; Kruger-Kjaer et al, 1998) to 20% using Hybrid Capture II (Clavel et al, 1999; Cuzick et al, 1999; Hill et al, 1999). Rates are about half these levels in women aged over 35 years. The rates for HPV type 16 (the most common oncogenic type) are much lower but, if only this type was used the sensitivity for high-grade CIN would be substantially reduced. However, judicious selection of a few HPV types might improve specificity without substantially affecting sensitivity.

### Modelling

A number of possibilities exist for introducing HPV testing at different ages and at different screening intervals. It could be used as the sole primary screening modality, as an adjunct to cytology, or in the triage of borderline and mild dyskaryosis. Published modelling studies are limited by the estimates of effectiveness, which are only now becoming available, and the cost of the test, which is still not known for high-volume applications. New modelling studies are presented based on the MISCAN micro-simulation programme (Habbema et al, 1984), using costs based on the British programme, and disease models based on the natural

 *© 2000 Cancer Research Campaign*

history of HPV-related cervical cancer. In the time available, only baseline calculations could be performed. These were sufficient to show that current knowledge is inadequate for assessing cost-effectiveness. The results of the modelling work show that for plausible values of prevalence, screening sensitivities and progression, HPV testing may be effective and cost-effective. For plausible assumptions about the model parameters, there are uses of HPV testing that would provide benefits at a lower cost than many existing healthcare programmes. However, the wide range of results that come from using high and low estimates for these parameters show that more data are needed to refine modelling using more accurate estimates of key parameters.

### Economic and social issues

A range of economic issues related to introducing HPV screening were surveyed as well as the very sparse literature on psychosocial aspects. In neither case is the database adequate to draw firm conclusions.

### CONCLUSIONS, IMPLICATIONS AND RECOMMENDATIONS

Ten conclusions were made in the report and each of these led to one or more recommendations, the latter being given in italics below.

1. The clearest role for HPV testing at the moment is in the management of women with borderline or mildly dyskaryotic smears (Kjellberg et al, 1998; Manos et al, 1999; Nobbenhuis et al, 1999; Solomon et al, submitted). In particular those aged over 30 who test positive for high-risk types could be referred immediately for colposcopy, while those younger than 30 years who test negative could receive less-intensive surveillance.

   *The evidence would support limited introduction with careful monitoring in this context. This should be done in such a way that comparisons with conventional management could be made.*

   *a. The safety of returning women with borderline/mild smears which are HPV negative to routine screening requires further research.*
   *b. HPV testing could either be performed on material stored from the initial scrape or by inviting women back for collection of a second sample. If a second sample is used it could either be taken shortly after the initial cytology result becomes available or at 6 months. The cost and psychological implications of these three alternatives requires careful evaluation.*

2. HPV testing with a consensus PCR method or Hybrid Capture II has a high sensitivity for high-grade CIN, usually exceeding that of cytology and certainly identifying cases missed by cytology.

   *Although this is not sufficient to recommend routine screening with HPV tests (see below), the evidence would appear to support limited use of the test in conjunction with cytology in certain situations (such as when women are likely not to return for further screening) where high sensitivity is important.*

   *Studies should be carried out to examine the safety of extending the screening interval and/or stopping screening after a certain age (e.g. 50 years) in women with history of negative results for both HPV and cytology.*

3. HPV testing appears to be less specific than cytology (as used for referral in the UK screening programme) with false-positive rates ranging from 3–10% in 'normal' women aged over 35 years: false-positive rates are higher in younger women. It should be noted that, if borderline smears are considered positive, the specificity of cytology is also poor particularly in younger women.

   *Further work is needed to clarify the management of HPV-positive but cytology-negative women and/or to establish methods for determining persistence of HPV infection.*

4. The potential exists for HPV testing using one of the newer assays to become the sole method of primary screening, especially for older women, but this will require consistent evidence of high sensitivity for high-grade lesions that are likely to progress to cancer. For younger women, cytology may only need to be performed in HPV-positive women. The cost-effectiveness of such an approach will depend largely on the relative cost of cytology and HPV testing and the screening interval employed.

   *More studies are needed.*

5. A full evaluation of HPV testing should provide information on the length of protection of a negative result and ideally demonstrate a reduction in cancer incidence. Several trials of current HPV tests in the 10 000 patient range are ongoing and should resolve issues of sensitivity, specificity and reproducibility and shed some light on the long-term risk of high-grade CIN following a negative HPV test.

   *a. New studies should take into account these ongoing trials.*
   *b. All large ongoing and future studies of HPV testing should follow women for at least 5 years.*
   *c. Ongoing studies should be encouraged to collaborate internationally to maximize the accuracy with which incidence reduction can be estimated.*
   *d. Consideration should be given to a very large (100 000–200 000 participants to receive HPV testing) randomized clinical trial to evaluate the effect of HPV testing on cancer incidence, and the length of protection afforded by a negative HPV test in conjunction with negative cytology. This should be incorporated into the national screening programme to minimize costs.*

6. A role may exist for HPV testing in post-treatment surveillance of high-grade CIN and localized cancer to determine more quickly and accurately if treatment has completely eradicated local disease. It may be possible to return women to routine surveillance if they become HPV-negative one year after treatment.

   *Focused trials in the area are needed.*

7. Modelling studies show that HPV testing may be a cost-effective screening modality either alone or in conjunction with cytology. These models are dependent on the input parameters, which are currently ill determined due to lack of data.

© 2000 Cancer Research Campaign

*In view of the lack of full evidence on the (cost-) effectiveness of HPV screening, it should not yet be implemented in routine primary screening. Further field studies are needed to improve estimates of the key model parameters.*

8. HPV testing has the potential to be used on self-collected cervical samples. This could help to improve coverage by reaching those who do not participate in the current screening programme (Wright et al, 2000).

   *a. The sensitivity of self-sampling requires further evaluation in settings appropriate to the NHS.*
   *b. Pilot work should investigate whether the option of self-sampling could improve coverage.*

9. HPV testing with Hybrid Capture appears to be a readily automatable procedure that can achieve high throughput with a low level of technical support. PCR methods using the MY09/11 or GP5+/6+ consensus primers provide good results, but are not yet commercially available.

   *More work is needed to evaluate the implementation of these assays in different laboratories.*

10. HPV testing needs to be viewed in the context of potential improvements in cytology using thin-layer preparations taken from liquid samples and automated reading.

   *A large study should evaluate the best way of integrating these technologies and the most cost-effective strategy.*

### REFERENCES

Bosch FX, Manos MM, Muñoz N, Sherman M, Jansen AM, Peto J et al (1995) Prevalence of human papillomavirus in cervical cancer: a worldwide perspective. International biological study on cervical cancer (IBSCC) Study Group. *J Natl Cancer Inst* **87**: 796–802

Burger MP, Hollema H, Pieters WJ et al (1996) Epidemiological evidence of cervical intraepithelial neoplasia without the presence of human papillomavirus. *Br J Cancer* **73**: 831–836

Clavel C, Masure M, Bory JP et al (1999) Hybrid Capture II-based human papillomavirus detection, a sensitive test to detect in routine high-grade cervical lesions: a preliminary study on 1518 women. *Br J Cancer* **80**: 1306–1311

Cox JT, Lorincz AT, Schiffman MH et al (1995) Human papillomavirus testing by hybrid capture appears to be useful in triaging women with a cytologic diagnosis of atypical squamous cells of undetermined significance. *Am J Obstet Gynecol* **172**: 946–954

Cuzick J, Szarewski A, Terry G et al (1995) Human papillomavirus testing in primary cervical screening. *Lancet* **345**: 1533–1537

Cuzick J, Beverley E, Ho L et al (1999a) HPV testing in primary screening of older women. *Br J Cancer* **81**: 554–558

Cuzick J, Sasieni P, Davies P et al (1999b) A systematic review of the role of human papillomavirus testing within a cervical screening programme. *Health Technol Assess* **3(14)**: 1–204

Cuzick J, Terry G, Ho L et al (2000) Association between high risk HPV types, HLA DRB1* and DQB1* alleles and cervical cancer in British women. *Br J Cancer* **82**: 1348–1352

de Roda Husman AM, Snijders PJ, Stel HV et al (1995) Processing of long-stored archival cervical smears for human papillomavirus detection by the polymerase chain reaction. *Br J Cancer* **72**: 412–417

Dillner J, Lehtinen M, Bjorge T et al (1997) Prospective seroepidemiologic study of human papillomavirus infection as a risk factor for invasive cervical cancer. *J Natl Cancer Inst* **89**: 1293–1299

Habbema JDF, Oortmarssen GJ van, Lubbe JThN et al (1984) The MISCAN simulation program for the evaluation of screening for disease. *Comput Methods Programs in Biomed* **20**: 79–93

Herrero R, Hildesheim A, Bratti C et al (2000) Population-based study of human papillomavirus infection and cervical neoplasia in rural Costa Rica. *J Natl Cancer Inst* **92**: 464–474

Herrington CS, Evans MF, Hallam NF et al (1995) Human papillomavirus status in the prediction of high-grade cervical intraepithelial neoplasia in patients with persistent low-grade cervical cytological abnormalities. *Br J Cancer* **71**: 206–209

Hill R, Kuhn L, Denny L et al (eds) (1999) Use of HPV DNA testing for cervical cancer screening: Results from the Khayelitsha Study, South Africa. In: *17th International Papillomavirus Conference*, Charleston, South Carolina

Ho GY, Burk RD, Klein S et al (1995) Persistent genital human papillomavirus infection as a risk factor for persistent cervical dysplasia. *J Natl Cancer Inst* **87**: 1365–1371

IARC Working Group on the Evaluation of Cervical Cancer Screening Programmes (1986) Screening for squamous cervical cancer: duration of low risk after negative results of cervical cytology and its implication for screening policies. *Br Med J* **293**: 659–664

IARC (1995) *IARC Monographs on the Evaluation of Carcinogenic Risks to Humans, Vol. 64. Human papillomaviruses.* International Agency for Research on Cancer: Lyon

ICRF Coordinating Committee on Cervical Screening (1984) Organisation of a programme for cervical cancer screening. *Br Med J* **289**: 894–895

ICRF Co-ordinating Committee on Cervical Screening (1986) The management of a cervical screening programme: a statement (October 1985). *Community Medicine* **3**: 179–184

Kjaer SK and Brinton LA (1993) Adenocarcinomas of the cervix: the epidemiology of an increasing problem. *Epidemiol Rev* **15**: 486–498

Kjellberg L, Wiklund F, Sjöberg I et al (1998) A population-based study of human papillomavirus deoxybribonucleic acid testing for predicting cervical intraepithelial neoplasia. *Am J Obstet Gynecol* **179**: 497–502

Kruger-Kjaer S, van den Brule AJ, Svare EI et al (1998) Different risk factor patterns for high-grade and low-grade intraepithelial lesions on the cervix among HPV-positive and HPV-negative young women. *Int J Cancer* **76**: 613–619

Lehtinen M, Dillner J, Knekt P et al (1996) Serologically diagnosed infection and human papillomavirus type 16 and risk for subsequent development of cervical carcinoma: nested case-control study. *Br Med J* **312**: 537–539

Manos MM, Wright DK, Lewis AJ et al (1989) The use of polymerase chain reaction amplification for the detection of genital human papillomaviruses. In: *Molecular Diagnostics of Human Cancer, Cancer Cells 7*, Furth M and Greaves M (eds), pp. 209–214. Cold Spring Harbor Press, NY

Manos MM, Kinney WK, Hurley LB et al (1999) Identifying women with cervical neoplasia: using human papillomavirus DNA testing for equivocal Papanicolaou results. *JAMA* **281**: 1605–1610

Nobbenhuis MAE, Walboomers JMM, Helmerhorst TJM et al (1999) Relation of human papillomavirus status to cervical lesions and consequences for cervical-cancer screening: a prospective study. *Lancet* **354**: 20–25

Olsen AO, Gjoen K, Sauer T et al (1995) Human papillomavirus and cervical intraepithelial neoplasia grade II/III: a population based case-control study. *Int J Cancer* **61**: 312–315

Peyton CL, Schiffman M, Lorincz AT et al (1998) Comparison of PCR and hybrid capture-based human papillomavirus detection systems using multiple cervical specimen collection strategies. *J Clin Microbiol* **36**: 3248–3254

Raffle AE, Alden B and Mackenzie EF (1995) Detection rates for abnormal cervical smears: what are we screening for? *Lancet* **345**: 1469–1473

Reid R, Greenberg MD, Lorincz A et al (1991) Should cervical cytologic testing be augmented by cervicography or human papillomavirus deoxyribonucleic acid detection? *Am J Obstet Gynecol* **164**: 1461–1471

Remmink AJ, Walboomers JM, Helmerhorst TJ et al (1995) The presence of persistent high-risk HPV genotypes in dysplastic cervical lesions is associated with progressive disease: natural history up to 36 months. *Int J Cancer* **61**: 306–311

Sasieni P, Cuzick J and Farmery E (1995) Accelerated decline in cervical cancer mortality in England and Wales. *Lancet* **346**: 1566–1567

Sasieni PD, Cuzick J, Lynch-Farmery E and the NCN Working Group (1996) Estimating the efficacy of screening by auditing smear histories of women with and without cervical cancer. *Br J Cancer* **73**: 1001–1005

Solomon D, Schiffman M, Tarone R for the ALTS Group. Comparison of HPV testing, cytology and immediate colposcopy in ASCUS triage: baseline review from randomized trial (ALTS). *J Natl Cancer Inst* (submitted)

Shafi MI (1994) Cytological surveillance avoids treatment. *Br Med J* **309**: 590–591

Vizcaino AP, Moreno V, Bosch FX et al (1998) International trends in the incidence of cervical cancer: I. Adenocarcinoma and adenosquamous cell carcinomas. *Int J Cancer* **75**: 536–545

Walboomers JM, Husman AM, Snijders PJ et al (1995) Human papillomavirus in false negative archival cervical smears: implications for screening for cervical cancer. *J Clin Pathol* **48**: 728–732

© 2000 Cancer Research Campaign

Walboomers JM, Jacobs MV, Manos MM et al (1999) Human papillomavirus is a necessary cause of invasive cervical cancer worldwide. *J Pathol* **189**: 12–19

Wright TC, Denny L, Kuhn L et al (2000) HPV DNA testing of self-collected vaginal samples compared with cytologic screening to detect cervical cancer. *JAMA* **283**: 81–86

*© 2000 Cancer Research Campaign*

## References

[PDF_00341] Bosch F. X., Manos M. M., Muñoz N., Sherman M., Jansen A. M., Peto J., Schiffman M. H., Moreno V., Kurman R., Shah K. V. (1995). Prevalence of human papillomavirus in cervical cancer: a worldwide perspective. International biological study on cervical cancer (IBSCC) Study Group.. J Natl Cancer Inst.

[PDF_00345] Burger M. P., Hollema H., Pieters W. J., Schröder F. P., Quint W. G. (1996). Epidemiological evidence of cervical intraepithelial neoplasia without the presence of human papillomavirus.. Br J Cancer.

[PDF_00348] Clavel C., Masure M., Bory J. P., Putaud I., Mangeonjean C., Lorenzato M., Gabriel R., Quereux C., Birembaut P. (1999). Hybrid Capture II-based human papillomavirus detection, a sensitive test to detect in routine high-grade cervical lesions: a preliminary study on 1518 women.. Br J Cancer.

[PDF_00351] Cox J. T., Lorincz A. T., Schiffman M. H., Sherman M. E., Cullen A., Kurman R. J. (1995). Human papillomavirus testing by hybrid capture appears to be useful in triaging women with a cytologic diagnosis of atypical squamous cells of undetermined significance.. Am J Obstet Gynecol.

[PDF_00357] Cuzick J., Beverley E., Ho L., Terry G., Sapper H., Mielzynska I., Lorincz A., Chan W. K., Krausz T., Soutter P. (1999). HPV testing in primary screening of older women.. Br J Cancer.

[PDF_00359] Cuzick J., Sasieni P., Davies P., Adams J., Normand C., Frater A., van Ballegooijen M., van den Akker E. (1999). A systematic review of the role of human papillomavirus testing within a cervical screening programme.. Health Technol Assess.

[PDF_00355] Cuzick J., Szarewski A., Terry G., Ho L., Hanby A., Maddox P., Anderson M., Kocjan G., Steele S. T., Guillebaud J. (1995). Human papillomavirus testing in primary cervical screening.. Lancet.

[PDF_00362] Cuzick J., Terry G., Ho L., Monaghan J., Lopes A., Clarkson P., Duncan I. (2000). Association between high-risk HPV types, HLA DRB1* and DQB1* alleles and cervical cancer in British women.. Br J Cancer.

[PDF_00368] Dillner J., Lehtinen M., Björge T., Luostarinen T., Youngman L., Jellum E., Koskela P., Gislefoss R. E., Hallmans G., Paavonen J. (1997). Prospective seroepidemiologic study of human papillomavirus infection as a risk factor for invasive cervical cancer.. J Natl Cancer Inst.

[PDF_00401] Dong Y. L., Gangula P. R., Fang L., Wimalawansa S. J., Yallampalli C. (1998). Uterine relaxation responses to calcitonin gene-related peptide and calcitonin gene-related peptide receptors decreased during labor in rats.. Am J Obstet Gynecol.

[PDF_00371] Habbema J. D., van Oortmarssen G. J., Lubbe J. T., van der Maas P. J. (1985). The MISCAN simulation program for the evaluation of screening for disease.. Comput Methods Programs Biomed.

[PDF_00374] Herrero R., Hildesheim A., Bratti C., Sherman M. E., Hutchinson M., Morales J., Balmaceda I., Greenberg M. D., Alfaro M., Burk R. D. (2000). Population-based study of human papillomavirus infection and cervical neoplasia in rural Costa Rica.. J Natl Cancer Inst.

[PDF_00377] Herrington C. S., Evans M. F., Hallam N. F., Charnock F. M., Gray W., McGee J. D. (1995). Human papillomavirus status in the prediction of high-grade cervical intraepithelial neoplasia in patients with persistent low-grade cervical cytological abnormalities.. Br J Cancer.

[PDF_00384] Ho G. Y., Burk R. D., Klein S., Kadish A. S., Chang C. J., Palan P., Basu J., Tachezy R., Lewis R., Romney S. (1995). Persistent genital human papillomavirus infection as a risk factor for persistent cervical dysplasia.. J Natl Cancer Inst.

[PDF_00399] Kjaer S. K., Brinton L. A. (1993). Adenocarcinomas of the uterine cervix: the epidemiology of an increasing problem.. Epidemiol Rev.

[PDF_00404] Krüger-Kjaer S., van den Brule A. J., Svare E. I., Engholm G., Sherman M. E., Poll P. A., Walboomers J. M., Bock J. E., Meijer C. J. (1998). Different risk factor patterns for high-grade and low-grade intraepithelial lesions on the cervix among HPV-positive and HPV-negative young women.. Int J Cancer.

[PDF_00408] Lehtinen M., Dillner J., Knekt P., Luostarinen T., Aromaa A., Kirnbauer R., Koskela P., Paavonen J., Peto R., Schiller J. T. (1996). Serologically diagnosed infection with human papillomavirus type 16 and risk for subsequent development of cervical carcinoma: nested case-control study.. BMJ.

[PDF_00415] Manos M. M., Kinney W. K., Hurley L. B., Sherman M. E., Shieh-Ngai J., Kurman R. J., Ransley J. E., Fetterman B. J., Hartinger J. S., McIntosh K. M. (1999). Identifying women with cervical neoplasia: using human papillomavirus DNA testing for equivocal Papanicolaou results.. JAMA.

[PDF_00418] Nobbenhuis M. A., Walboomers J. M., Helmerhorst T. J., Rozendaal L., Remmink A. J., Risse E. K., van der Linden H. C., Voorhorst F. J., Kenemans P., Meijer C. J. (1999). Relation of human papillomavirus status to cervical lesions and consequences for cervical-cancer screening: a prospective study.. Lancet.

[PDF_00421] Olsen A. O., Gjøen K., Sauer T., Orstavik I., Naess O., Kierulf K., Sponland G., Magnus P. (1995). Human papillomavirus and cervical intraepithelial neoplasia grade II-III: a population-based case-control study.. Int J Cancer.

[PDF_00424] Peyton C. L., Schiffman M., Lörincz A. T., Hunt W. C., Mielzynska I., Bratti C., Eaton S., Hildesheim A., Morera L. A., Rodriguez A. C. (1998). Comparison of PCR- and hybrid capture-based human papillomavirus detection systems using multiple cervical specimen collection strategies.. J Clin Microbiol.

[PDF_00427] Raffle A. E., Alden B., Mackenzie E. F. (1995). Detection rates for abnormal cervical smears: what are we screening for?. Lancet.

[PDF_00429] Reid R., Greenberg M. D., Lorincz A., Jenson A. B., Laverty C. R., Husain M., Daoud Y., Zado B., White T., Cantor D. (1991). Should cervical cytologic testing be augmented by cervicography or human papillomavirus deoxyribonucleic acid detection?. Am J Obstet Gynecol.

[PDF_00432] Remmink A. J., Walboomers J. M., Helmerhorst T. J., Voorhorst F. J., Rozendaal L., Risse E. K., Meijer C. J., Kenemans P. (1995). The presence of persistent high-risk HPV genotypes in dysplastic cervical lesions is associated with progressive disease: natural history up to 36 months.. Int J Cancer.

[PDF_00438] Sasieni P. D., Cuzick J., Lynch-Farmery E. (1996). Estimating the efficacy of screening by auditing smear histories of women with and without cervical cancer. The National Co-ordinating Network for Cervical Screening Working Group.. Br J Cancer.

[PDF_00436] Sasieni P., Cuzick J., Farmery E. (1995). Accelerated decline in cervical cancer mortality in England and Wales.. Lancet.

[PDF_00444] Shafi M. I. (1994). Management of women with mild dyskaryosis. Cytological surveillance avoids overtreatment.. BMJ.

[PDF_00445] Vizcaino A. P., Moreno V., Bosch F. X., Muñoz N., Barros-Dios X. M., Parkin D. M. (1998). International trends in the incidence of cervical cancer: I. Adenocarcinoma and adenosquamous cell carcinomas.. Int J Cancer.

[PDF_00453] Walboomers J. M., Jacobs M. V., Manos M. M., Bosch F. X., Kummer J. A., Shah K. V., Snijders P. J., Peto J., Meijer C. J., Muñoz N. (1999). Human papillomavirus is a necessary cause of invasive cervical cancer worldwide.. J Pathol.

[PDF_00448] Walboomers J. M., de Roda Husman A. M., Snijders P. J., Stel H. V., Risse E. K., Helmerhorst T. J., Voorhorst F. J., Meijer C. J. (1995). Human papillomavirus in false negative archival cervical smears: implications for screening for cervical cancer.. J Clin Pathol.

[PDF_00455] Wright T. C., Denny L., Kuhn L., Pollack A., Lorincz A. (2000). HPV DNA testing of self-collected vaginal samples compared with cytologic screening to detect cervical cancer.. JAMA.

[PDF_00365] de Roda Husman A. M., Snijders P. J., Stel H. V., van den Brule A. J., Meijer C. J., Walboomers J. M. (1995). Processing of long-stored archival cervical smears for human papillomavirus detection by the polymerase chain reaction.. Br J Cancer.

